# Application of Sorbents for Oil Spill Cleanup Focusing on Natural-Based Modified Materials: A Review

**DOI:** 10.3390/molecules25194522

**Published:** 2020-10-02

**Authors:** Miltiadis Zamparas, Dimitrios Tzivras, Vassilios Dracopoulos, Theophilos Ioannides

**Affiliations:** 1 Institute of Chemical Engineering Sciences, Foundation for Research and Technology-Hellas, 26504 Patras, Greece; dimitristzvrs@gmail.com (D.T.); indy@iceht.forth.gr (V.D.); 2 Department of Chemistry, University of Patras, 26504 Patras, Greece

**Keywords:** oil spill cleanup, sorbent, lignocellulosic materials, modification methods, bio-based aerogels

## Abstract

Conventional synthetic sorbents for oil spill removal are the most widely applied materials, although they are not the optimal choices from an economic and environmental point of view. The use of inexpensive, abundant, non-toxic, biodegradable, and reusable lignocellulosic materials might be an alternative to conventional sorbents, with obvious positive impact on sustainability and circular economy. The objective of this paper was to review reports on the use of natural-based adsorbing materials for the restoration of water bodies threatened by oil spills. The use of raw and modified natural sorbents as a restoration tool, their sorption capacity, along with the individual results in conditions that have been implemented, were examined in detail. Modification methods for improving the hydrophobicity of natural sorbents were also extensively highlighted. Furthermore, an attempt was made to assess the advantages and limitations of each natural sorbent since one material is unlikely to encompass all potential oil spill scenarios. Finally, an evaluation was conducted in order to outline an integrated approach based on the terms of material–environment–economy.

## 1. Introduction

Nowadays, the rising number of accidents and oil leakages has led to the continuous improvement of oil spill cleaning techniques in order to address them immediately, minimizing their disastrous effects [1,2]. This need is intensified by the fact that oil spill pollution is one of the most visible examples of water quality degradation due to human activities, affecting aquatic ecosystems worldwide [3,4]. Notable cases are the “Prestige” shipwreck (NA Atlantic) in 2002 [5], “Sea Diamond” shipwreck (Santorini) in 2007 [6], Deep Horizon (Gulf of Mexico) in 2010 [3], and “Agia Zoni II” (Saronic Gulf) in 2017 [4]. On the 10th of September 2017, the vessel Agia Zoni II sank in the Saronic Gulf, Greece, releasing an estimated 2.500 tons of crude oil, which profoundly contaminated the coasts of Salamina and the Athens Riviera [7].

**Figure 1:**
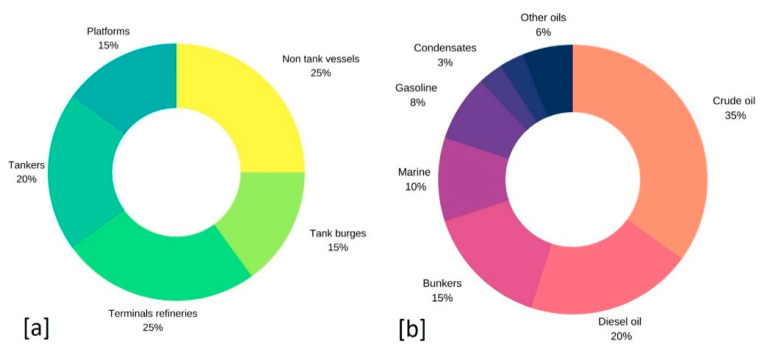
(**a**) Percentage (%) of origin of oil spills in the water. (**b**) Percentage (%) of oil types (authors’ own study, data collected from [2]).

Oil disperses very quickly in water, forms a slick on the water surface, and progressively submerges and accumulates in the sediments. Currents and wind tend to cause an even faster distribution, leading to degradation and even the extinction of vast expanses of marine life and environment [8,9]. In combination with the above conditions, a variety of physicochemical parameters such as temperature and salinity may also determine the spread rates of oil spills in a natural water body [10,11]. Figure 1 shows that the most significant volume of oil spilled into water derives from vessels (25%), tankers (20%), and tank barges (15%), while an additional percentage comes from refinery terminals (25%) and platforms (15%). The most typically encountered oil types are crude oil (35%), diesel oil (20%), marine oil (10%), gasoline (8%), and oil condensates (3%).

**Figure 2:**
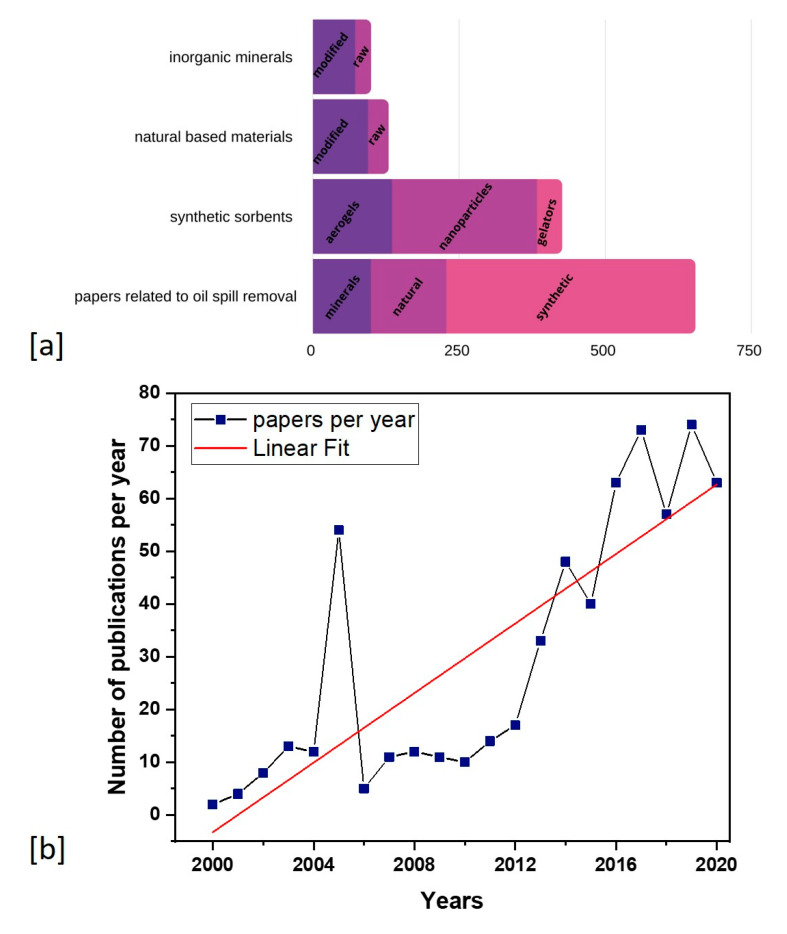
(**a**) Publications related to oil spill removal separated into basic categories. (**b**) Publications related to oil spill per year from 2000 to 2020. The linear regression line shows a rising trend of publications related to oil spill cleanup during the study period of the last 20 years (authors’ own study, data from Scopus).

Once the oil becomes dispersed on the water surface, it undergoes a number of processes, such as spreading, evaporation, dissolution, biodegradation, emulsification, photo-oxidation, sinking, and tarball formation, making cleanup highly complex [12]. For these reasons, it is crucial to remove oil spills as quickly as possible. A literature overview indicates that a variety of materials have been examined extensively for this purpose and can be categorized into (a) inorganic mineral sorbents [13,14,15,16,17], (b) synthetic organic sorbents [18,19,20,21,22], and (c) natural organic sorbents [23,24,25] (Figure 2). The large number of published articles (from 2000 to 2020) addressing oil spill cleanup indicates the current growing attention being paid to environmental protection (Figure 2). Moreover, the linear regression line shows a rising trend during the study period of the last 20 years, revealing the growing interest of many researchers in this field. Undoubtedly, sorption is an efficient method in which the aforementioned materials absorb oil in amounts multiple times their self-weight, transforming it into a solid or semi-solid state for appropriate disposal or reuse. Several studies have been carried out on the sorption of oil by oleophilic–hydrophobic synthetic materials (Figure 2). The major drawback of these materials is the extensive chemical modification that they have undergone, making them economically and environmentally non-friendly. More specifically, some materials can form hazardous species or low molecular weight organic complexes, bioavailable for aquatic organisms [26]. Besides, mineral sorbents have a severe shortcoming, as the majority of them sink into the water column, contaminating the seabed. They also usually leach some of the oil, while precipitating, due to their low retention capability [26].

Currently, there is growing interest in finding inexpensive, abundant, and effective materials as oil spill sorbents in water, focusing on natural organic sorbents mainly from agriculture (Figure 2). A wide variety of cellulosic materials such as banana fibers [27], corn stalk [25], cotton [28], milkweed fiber [29] curauna fibers [30], kapok [31], luffa [32], nettle fibers [33], orange peel [34], palm fibers [35], pineapple leaves [36], pistia leaves and roots [37], pomelo peel [38], rice husks [39], sawdust [40], wheat straw [41], sugarcane bagasse [42], and walnut shell [43] have shown good results, making them potential candidates for oil spill treatment.

Although natural-based materials are low cost, abundant, eco-friendly, and support proper utilization of waste, their sorption efficiency is inferior compared to some synthetic materials. Their main disadvantages stem from their poor oleophilic/hydrophobic properties. In order to improve these characteristics, the sorbents can be modified using mechanical [44], thermal [45], and chemical [46] modification methods. Mechanical modification methods affect the adsorption capacity but do not improve the hydrophobicity of the adsorbent. On the other hand, the use of thermal [47], hydrothermal [48], and also chemical modification methods such as mercerization [49], acetylation [50], benzoylation [51], and grafting [52] may improve significantly the sorption properties of natural-based materials.

The objective of this paper was to review the restoration methods of degraded waters threatened by oil spills, emphasizing lignocellulosic adsorbing materials. The use of raw and modified natural sorbents as a restoration tool, their sorption capacity, as well as the individual results in conditions that have been implemented were examined in detail. Modification methods for improving the hydrophobicity of natural sorbents were also extensively highlighted. Furthermore, an attempt was made to assess the advantages and limitations of each natural sorbent since one material is unlikely to be fitting in all potential oil spill scenarios. Finally, an evaluation was conducted in order to outline an integrated approach based on the terms of material–environment–economy.

## 2. The Liquid–Solid Interface between Oil and Sorbent Materials

**Figure 3:**
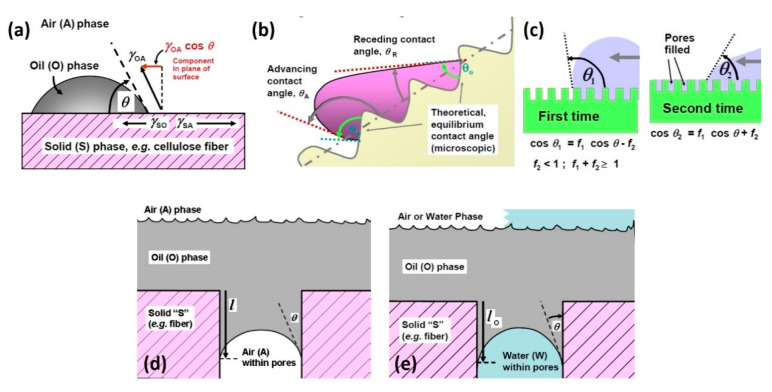
Processes taking place at the liquid–solid interface between oil and sorbent materials. (**a**) Wettability of oil on the surface of a sorbent, (**b**) advancing and receding angles on a rough surface, (**c**) Cassie and Baxter’s theory relative to initial and following wetting of a porous surface, (**d**) penetration of oil into an idealized pore of a dry sorbent, (**e**) penetration of oil into an idealized pore of a water-wet sorbent (adapted and modified from [55]).

Surface chemical state and morphology define surface wettability, which strongly correlates with the oil spill sorption. The contact angle is applied to evaluate wettability. In case the contact angle of a droplet on a smooth surface is less than 90°, it is categorized as wettable [53]. If the contact angle is higher than 90°, the liquid will avoid diffusing, and the material is classified as non-wettable by the liquid (Figure 3a). In case the liquid is oil, the sorbent can be categorized as oleophilic and oleophobic, respectively. Similar measurements can be conducted for water droplets, and the sorbent can be likewise categorized as hydrophilic or hydrophobic [53]. The hydrophobicity of the material surface blocks water adsorption and hence boosts oil sorption efficiency because of the absence of competition between water and oil molecules. Under realistic conditions, several factors interact to prevent the initial wetting of a surface, which also tends to oppose the retraction of fluid after wetting has taken place [54]. The roughness and heterogeneity of real surfaces can alter contact angles and wettability with advancing and receding sites on the surface (Figure 3b). Furthermore, in the beginning, during the gradual wetting of the surface, the existing pores do not cause any attractive force on the nearing liquid phase. Contrariwise, when the surface has been saturated by a liquid, voids will tend to remain loaded, impacting the interfacial force [54].

Additionally, numerous publications mention that capillary action is a crucial process via which a porous structure holds oil, and contact angle phenomena control the flow rate of petroleum into porous substrates (Figure 3d,e). Figure 3d,e take into account the condition whereby a dry and a water-saturated, porous substrate are applied for oil spill cleanup. Water presence in the sorbent can alter the contact angle, and such a change will not be beneficial for oil removal by a natural-based material.

## 3. Modification Methods for Hydrophobicity Improvement

### 3.1. Physical and Thermal Treatment

Natural sorbent media that are grinded show high oil sorption capacity per unit mass, due to better accessibility with the contact surface of the material, with binding sites being more available on smaller particles. Several articles confirm this general observation relating natural-based materials to oil spill removal. For example, Bayat et al. [56] ground up bagasse—a byproduct of sugar extraction from sugarcane—and tested samples with varying particle sizes. Experiments were conducted with samples having different fraction sizes of bagasse (from 14 to 45 mm) to verify the effect of particle size on oil spill cleanup treatment. The results reveal that oil removal efficiency rises with a decrease in particle size [56]. Likewise, Husseien et al. [45] noted the highest sorption capacity for oil when using fibrous bio-sorbents such as corn stalk in the form of fine fibers.

**Figure 4:**
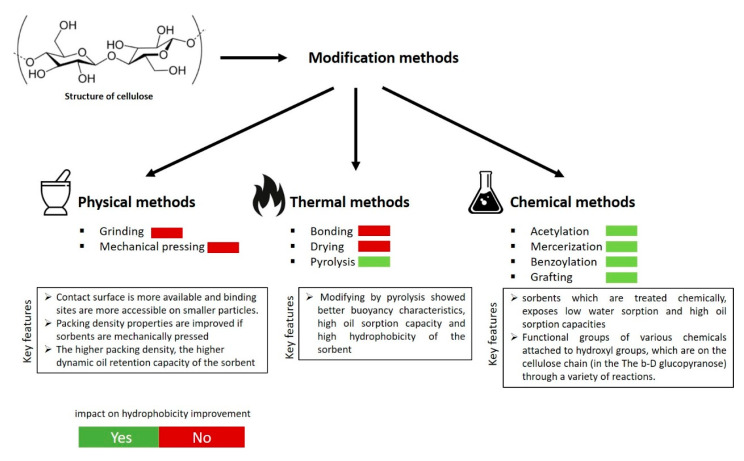
Classification of modification methods for hydrophobicity improvement in natural-based sorbents (authors’ own study).

Thermal treatment at low temperatures, i.e., drying, does not affect the oleophilic and hydrophobic properties of the sorbent (Figure 4). Its main effect is to remove some impurities of the surface, allowing easier adsorption of oil [34]. On the other hand, a high-temperature thermal treatment like pyrolysis [57] leads to the carbonization of the sorbent. Thus, the oil sorption capacity and oil to water selectivity are highly enhanced [58] (Figure 4).

Vlaev et al. [59] reported on BRHA (brown rice husk ash) and WRHA (white rice husk ash) functionalized by pyrolysis. The new products showed a sorption capacity of 5.02 and 6.22 g/g (BRHA) and 2.78 and 2.98 g/g (WRHA) for diesel and crude oil, respectively. Besides, BRHA showed better hydrophobic and buoyancy characteristics than WRHA. Kudaybergenov et al. [60] demonstrated thermally treated rice husk (heating under CO_2_ at 800 °C) having a sorption efficiency of ~15 g/g for crude oil. Uzunov et al. [61] examined the impact of pyrolysis at temperatures between 250 and 700 °C on crude oil sorption capacity. Rice husk pyrolyzed at 350 °C showed a maximum sorption capacity of ~10 g/g, in comparison with raw rice husk (~6.5 g/g).

**Figure 5:**
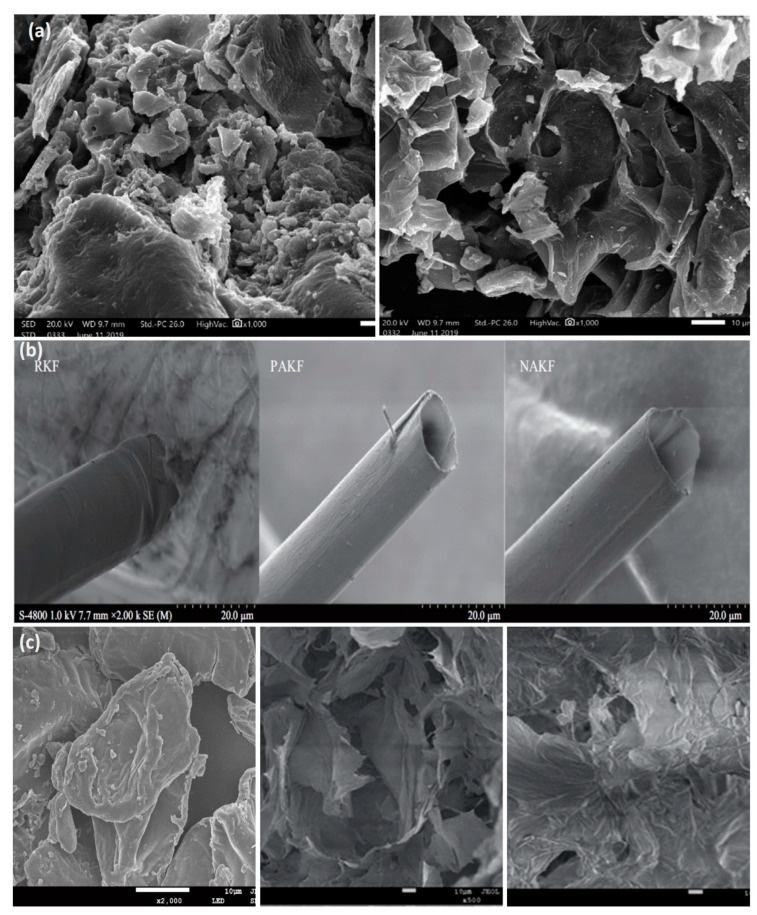
SEM images of (**a**) raw orange peel (OP) and orange peel thermally modified (TMOP) at 500 °C (adapted from [34] with permission from Elsevier), (**b**) of raw kapok fiber (RKF), pyridine-catalyzed kapok fiber (PAKF), and NBS-catalyzed kapok fiber (NAKF) (adapted from [82] with permission from Elsevier), (**c**) raw pomelo peel, pomelo sponge aerogels (PSA), and hydrophobic sponge aerogels (HPSA) with MTMS (adapted from [86] with permission from RS).

In the same study, the authors assumed that the capacity of the pyrolyzed rice husk on oil sorption is affected by the porous substrate instead of the impact of the surface functional groups [61]. Furthermore, the oil sorption efficiency of pyrolyzed rice husk was studied in a similar work [58]. The authors examined the capacity of rice husk pyrolyzed at 480 °C on oil sorption. The capacities of the studied material on different forms of oil follow the order of heavy crude oil > motor oil > light crude oil > diesel > gasoline, showing that the permeation of the oils into the sorbent reduced with the increase in bulk density [58]. Husseien et al. [62] evaluated the sorption properties of barley straw pyrolyzed at temperatures between 200 and 500 °C. Oil sorption capacity was found to be 7.6 g/g and 9.2 g/g for diesel and heavy oil, respectively (Table 1). Carbonized barley straw applied as a pad indicated highly hydrophobic characteristics, adsorbing oil at more excessive amounts than polypropylene commercial pads (Table 1). Moreover, they were reusable for two absorption/desorption cycles [62]. Τhe surface alteration of thermally treated natural-based materials is reflected in the SEM images of El Gheriany’s study [34]. The SEM images (Figure 5a) revealed that the originally smooth and homogeneous structure of the raw orange peel appeared rough and of high porosity after thermal decomposition at 500 °C. The occurrence of pores is ascribed to material decomposition and desorption of gases/vapors during pyrolysis (Figure 5a).

### 3.2. Hydrothermal Treatment

Hydrothermal treatment is an alternative to thermal pyrolysis for the production of carbonaceous materials. It is one of the most widely used treatment methods in sub- or supercritical water conditions [63,64]. Hydrothermal treatment diminishes the oxygen-containing functional groups and volatile elements, enhancing the carbon content of the natural sorbents [64]. Consequently, the hydrophobicity of the prepared cellulosic materials is significantly improved. Hydrothermal treatment is advantageous, especially for the fabrication of sorbents derived from natural biomass [48,64]. Several types of biomass like wood, plants, and vegetables have a high water content that may exceed 70% of their total weight. Thus, simple thermal techniques can be expensive and time-consuming because of the necessity of material drying before modification in order to lower the water content. On the contrary, hydrothermal treatment allows the thermal modification of the natural material in solution. Therefore, the energy and time-consuming step of drying is avoided [65,66,67,68]. Li et al. studied the efficiency of winter melon aerogel on oil sorption made by a hydrothermal process [68]. The aerogel had a density of 0.048 g/cm^3^ and adsorption capacity of 25 g/g. The natural-based aerogel showed remarkable hydrophobicity (water contact angle 135°), low density, and high porosity, enhancing the oleophilic properties [69].

### 3.3. Chemical Modification Methods

#### 3.3.1. Mercerization

Cellulose is a polymer with β-glucose as its structural units, which are joined together by β-1,4 glycosidic bonds. The molecular weight of cellulose varies depending on its origin. Each glucose structural unit contains three free hydroxyl groups, two secondary and one primary. Cellulose is found in more than one crystalline form, with the most important being cellulose I and cellulose II [70]. The cellulose surface is quite hydrophilic, which is not advantageous for efficient oil–water separation, as hydrophobic and oleophilic characteristics are both needed. Cellulose I is found in natural fibers [71]. Forcible swelling of the cotton, e.g., treated with NaOH (mercerization), changes the crystal lattice and converts cellulose I to cellulose II. Specifically, mercerization is an alkali treatment of the sorbent’s fibers with hot or cold NaOH solution leading to the removal of natural and artificial impurities. Moreover, the fibers do not change their form, but they change their crystallinity from cellulose I to cellulose II. Thus, the surface of the material becomes rough and rigid, increasing the contact area, which leads to higher and more effective adsorption [72,73]. Morphological information regarding the effects of mercerization on sawdust was derived by Gulati et al. [74]. SEM shows the smooth surface of the raw sawdust and the substantial change in the morphology of the mercerized new material [74].

Modification of the surface will provide better contact between material and oil. Thus, mercerized cellulose attains properties that make it favorable for oil spill removal from water media [74]. According to Wong [49], mercerization increased the crude oil sorption capacity of soybean residue up to 5 g/g and the motor oil sorption capacity of cattail fibers up to 4 g/g (Table 1). Moreover, rice husks have been applied as feedstock for the fabrication of mercerized natural-based oil sorbents [75]. According to Bazargan [75], alkali treatment was shown to alter the rice husk structure, creating a material with adequate oil sorption capacity (19 g/g). The suggested method occurs at low temperatures, resulting in high product yields. BET (Brunauer–Emmett–Teller) and FTIR (Fourier Transform Infrared) analyses have indicated that microporosity, along with surface functional groups, are not the main controlling factors in the oil sorption capacity. The marine diesel uptake capacity was shown to have a strong inverse relationship with the bulk density [75]. The modified rice husk exhibits a reduced bulk density, which permits oil to diffuse internally into the substrate [75].

#### 3.3.2. Acetylation

Acetylation is a technique for increasing the sorption efficiency of the sorbent and can be facilitated by the use of a catalyst (*N*-bromosuccinimide, *N*-methylpyrrolidone, 4-dimethylaminopyridine) or not. In acetylation, the hydroxyl groups in the cellulose structure are converted to oleophilic acetate groups (O-CO-CH_3_) through reaction with acetic anhydride, lowering the hydrophilic properties of the sorbent and enhancing the oleophilic ones [46,50,76,77,78]. As a result of this modification, the oil uptake is drastically increased, while the water sorption is respectively reduced. Acetylated sorbents have bulkier forms because of the difference in molecular weight between OH and O-CO-CH_3_, which is referred to as weight percent gain [46,78].

Many studies have revealed its efficiency in increasing the hydrophobicity of natural fibers. In one of them, the usage of acetylated rice straw produced through catalytic and non-catalytic processes was examined [77]. Acetylation, without the presence of a catalyst, led to an increase in sorbent weight of 11.2% and to oil sorption efficiency in the range of 16.8–20 g/g. Acetylation in the presence of a catalyst (4-dimethyl-aminopyridine) indicated a slightly higher increase in sorbent weight (15.4%), with sorption ranging between 20.9 and 24 g/g. It is noteworthy to mention that the sorption efficiency of the acetylated rice husk increases pro rata with the degree of acetylation [77]. Deschamps et al. [79] examined the efficiency of unmodified and acetylated cotton fibers on the sorption of different oil forms (fuel, crude, mineral, vegetable) from aqueous solutions. In this study, oil uptake capacities ranged from 19 to 23 g/g and 23 to 30 g/g for raw and treated cotton, respectively. Moreover, the material could be reused by simple mechanical pressing for up to 10 times. As shown therein, after the 10th cycle, sorption efficiencies decreased from 15 and 21 g/g to 11.5 to 12.5 g/g for raw and acetylated cotton, respectively. Remarkably, the treated fibers sorb smaller amounts of oil compared to the unmodified ones. Nevertheless, they exhibited stronger lipophilic properties and better stability in the aqueous solution during the sorption experiments. Furthermore, Hussein et al. [80] pointed out that cotton fiber in the form of loose fiber or pad displayed good results for oil spill removal, with sorption efficiencies varying between 18.43 and 22.5 g/g. A subsequent study by Li et al. [81] focused on the characterization of acetylated corn straw fibers in aqueous solutions containing crude, diesel, and vacuum pump oil, respectively. The results showed that the acetylated cellulose fiber, an ultra-oleophilic material, revealed uptake capacities of 67.54, 52.65, and 42.53 g/g for crude oil, diesel oil, and vacuum pump oil, respectively. It is also noteworthy that modified material floated on the oil–water surface for several days without sinking. Furthermore, the water contact angle was 51.1° and 120.65° for the unmodified and acetylated fiber, respectively. Teli and Valia [50] modified coconut fiber via acetylation at 100 °C with a NBS catalyst (1%). Adsorption efficiencies of 3.5 g/g for the unmodified coconut coir were enhanced to 15.75 g/g for the acetylated material. Moreover, Asadpour et al. [76] studied the efficiency of the acetylated oil palm fibers on oil spill removal. The BET surface area of oil palm fibers was found to be 0.40 and 0.35 m^2^/g for raw and modified fibers, respectively. The acetylated fibers reached a maximum uptake capacity of 6.8 and 7.0 g/g for tapis and arabian crude oils, respectively. Ιn addition, they achieved buoyancy in the order of 93.7% and 95.3%, respectively.

Moreover, the acetylation method was used to enhance the hydrophobicity of kapok fibers. Kapok is considered one of the best natural-based materials, with high oil sorption efficiency and excellent buoyancy characteristics. According to Wang et al. [82], kapok fibers were effectively acetylated, and the subsequent fibers adsorbed a greater amount (36.7 g/g) than raw fibers (27 g/g) for diesel oil. The nonpolar nature of acetyl groups makes the surface of modified kapok fibers hydrophobic. Consequently, a significant amount of oil was favorably absorbed into the fibers, with small volumes of water retained on them. The reported results can be correlated with the SEM images (Figure 5b) of raw and modified kapok fibers. Raw kapok has a hollow shape and smooth surface with a closed orifice. At the same time, acetylated materials PAKF and NAKF show a tiny groove on the surface and an open lumen orifice. This implies that the smooth surface (raw material) is less beneficial for oil sorption, and the improved new materials with a rough surface are favorable for oil sorption [82].

#### 3.3.3. Grafting

Polymerization is an effective and straightforward process to graft monomers onto the fiber surface [83]. This method has been applied by researchers both for synthetic and natural-based sorbents, such as polypropylene [84], banana [52], and coir [85]. The aforementioned studies indicated that the maximum oil sorption capacity of grafted banana fibers was 14.45 g/g [52] and for grafted coir 13.45 g/g [85]. Banana fibers are grafted to form esters, replacing the hydroxyl group in the cellulose surface [52]. Nevertheless, the percentage of the synthetic polymer chain in the new grafted material was controlled in order to not interfere with swelling of separated fibers in water. This factor is crucial in a grafted natural-based sorbent for removing emulsified oil from water. The surface structure of banana fiber and grafted banana fiber was evaluated using SEM. Images from SEM indicated that there was a substantial alteration between the surface of raw and modified banana fiber. Chemically grafted banana fiber was coated with poly butyl acrylate species in a heterogeneous way on the surface. Moreover, some fibers were cross-linked together as a result of the reaction. Thus, the fiber becomes bulkier and hydrophobic, giving high absorptivity toward oil [52].

In addition, Viju et al. pointed out the significance of the grafting method in determining the oil sorption efficiency of nettle fibers [83]. The maximum oil uptake capacity of grafted nettle was 36.60 and 25.56 g/g for crude oil and vegetable oil, respectively (Table 1). Reusability experiments revealed that modified nettle showed better oil uptake capacity than raw nettle fibers following seven sorption–desorption cycles (Table 1). An important finding was that modified nettle had higher oil uptake capacity than a commercial polypropylene material [83].

Natural rubber (NR) foam was modified via graft copolymerization with an oleophilic monomer such as methyl methacrylate (MMA). NR foam was successfully modified by graft copolymerization with Poly(methyl methacrylate) (PMMA) [87]. The maximum oil sorption capacity of gasoline, diesel, engine oil, toluene, and xylene were 9.9, 8.6, 6.0, 11.8, and 10.9 g/g, respectively. The oil absorbency of PMMA-NR foam was higher than the unmodified NR foam (6.7, 7.0, 2.5, 9.6, 9.7 g/g) [87]. Furthermore, the capability of raw and grafted sugarcane bagasse in oil sorption was investigated by Said et al. [88]. The chemical modification was performed by grafting raw sugarcane bagasse with fatty acid adding hydrophobic properties to the bagasse matrix. Τhe attained oil uptake capacity for the grafted bagasse was 3 g/g.

## 4. Bio-Based Aerogels for Oil Spill Cleanup

**Figure 6:**
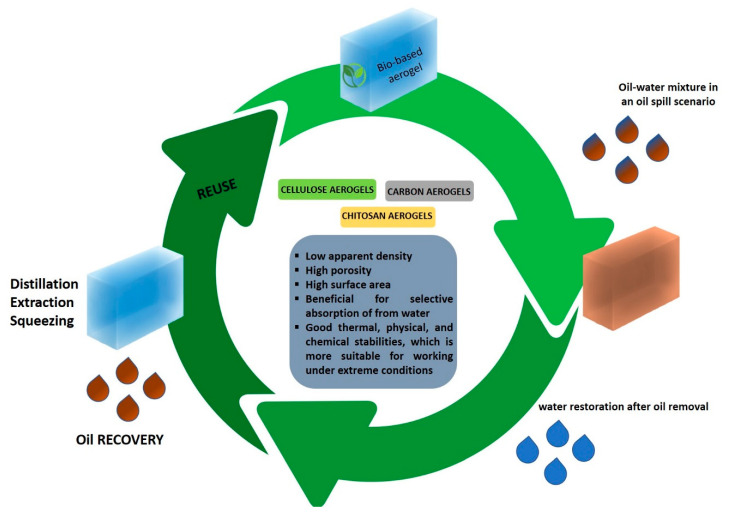
Natural-based aerogels for removing oil emulsified from water.

**Figure 7:**
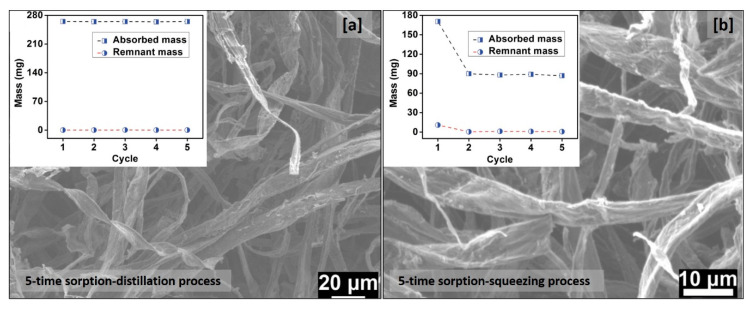
(**a**) Effect of cycles on oil sorption capacity of the CMB aerogel and SEM image after 5-time sorption/distillation process. (**b**) Effect of cycles on oil sorption capacity of the CMB aerogel and SEM image after 5-time sorption/squeezing process (modified from [94]).

The main advantage of aerogels for oil spill cleanup is the recovery of the adsorbed oil and the reusability/recyclability of the sorbent (Figure 6). Several types of recovery methods have been described, such as extraction [89], distillation [90], and squeezing [91]. Combustion has also been reported as a way to recover the absorbent but not the oil [92]. Mechanical squeezing (Figure 7) is the most environmentally friendly method to retrieve the oil, compared to the other methods, following the order of squeezing–distillation–extraction. Extraction is the least eco-friendly recovery method in which organic solvents have been used to extract oil, producing an oil/solvent mixture that has to be further processed.

However, the stability of the sorbents in terms of their mechanical and chemical properties is crucial for their reuse. Mechanical pressure during the recovery of oil might cause deformations in the pore structure of the sorbent, resulting in decreased oil sorption capacity. Moreover, the lignocellulosic materials should be stable in the presence of elevated temperatures and solvents during oil recovery by distillation and/or extraction, respectively [93].

Two of the above methods were presented in the study of Bi et al. [94]. The recovery tests were performed for carbon microbelt (CMB) aerogel through distillation and squeezing. At first, oil was sorbed by the CMB aerogel. After this, the material was heated to release the oil vapor. This sorption–distillation process was repeated for up to five cycles. As shown in Figure 7a, less than 1 wt% of residual oil remained in the CMB aerogel after each cycle, and no obvious change in sorption capacity was observed after five cycles, showing the steady sorption and recycling efficiency of the CMB aerogel [94]. Moreover, according to the SEM image, no structural damage was observed to the material after five cycles of liquid recovery (Figure 7a).

Figure 7b shows the oil recovery test through squeezing. In the first cycle, 170 mg of the oil was sorbed by the CMB aerogel, but the residue mass was up to 10.8 mg after squeezing. From the second cycle onwards, the of CMB aerogel became stable, i.e., the weight gain remained constant; however, the oil adsorption capacity was significantly reduced (from 170 to 90 mg) [94].

**Figure 8:**
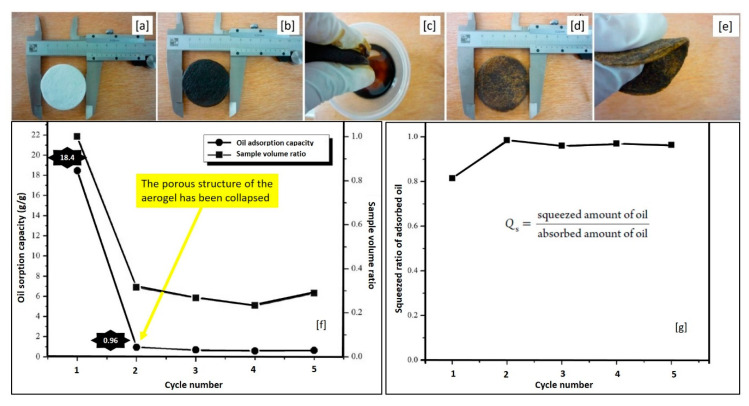
The squeezing process. (**a**) Cellulose aerogel from paper waste, (**b**) cellulose aerogel after first sorption cycle, (**c**) mechanical squeezing, (**d**) cellulose aerogel sample after squeezing, (**e**) good flexibility of the material, (**f**) effect of cycles on oil sorption capacity and sample volume of the aerogel, (**g**) effect of cycles on squeezed ratio of absorbed oil (adapted and modified from [95] with permission from ACS).

In another study, the impact of squeezing on the oil absorption capacity of a bio-based aerogel from paper waste was investigated by Nguyen et al. [95]. Figure 8a,b depict the cellulose aerogel sample before and after the first oil sorption cycle. The sizes of the material were almost unchanged after oil sorption. To recover the absorbed crude oil, a simple mechanical squeezing was performed (Figure 8c). In addition, Figure 8d shows the material after squeezing, where its shape had not changed significantly, and Figure 8e illustrates the good flexibility of the aerogel after oil recovery [95]. Afterwards, the utilized material was reused for the next absorption test cycle.

The material reached a high absorption capacity of 18.4 g/g in the first cycle, but the capacity dropped dramatically during the following cycles. This phenomenon can be explained based on the change in the cellulose aerogel volume (Figure 8f). After the first cycle, the ratio of the volume of the squeezed sample and its original volume was 0.32, indicating that the porous structure of the aerogel largely collapsed (Figure 8f). Consequently, the oil absorption capacity of the aerogel sharply decreased to 0.96 g/g after the second cycle. In the next cycles, the volume ratio values were similar to the first value, indicating that the material structure did not alter anymore [95]. Regarding the squeezed amount of the absorbed oil (calculated by the equation presented in Figure 8g), 81.5, 98.5, 95.9, 96.9, and 96.4% of the absorbed oil was released after the 1st, 2nd, 3rd, 4th and 5th cycle respectively.

### Super-Hydrophobic Bio-Based Aerogels

**Figure 9:**
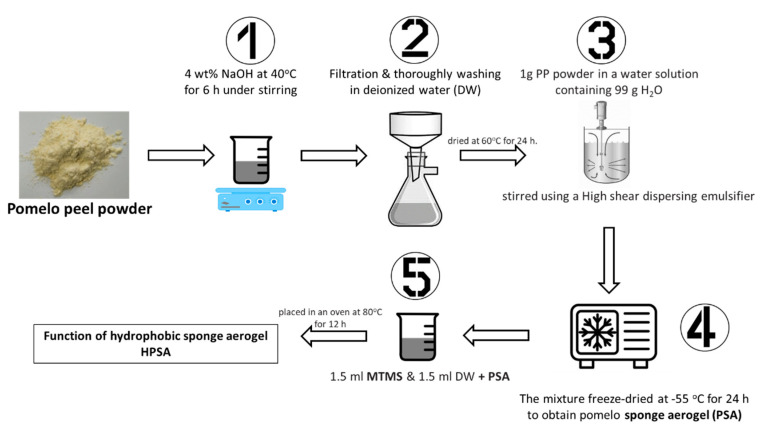
Preparation of hydrophobic sponge aerogels based on pomelo peel powder (based on information from [85]).

Several cellulose-based materials were modified by methyltrimethoxysilane (MTMS) as the modification reagent, achieving excellent hydrophobicity and operational stability [96,97]. For instance, Feng et al. [98] utilized MTMS on cellulose fibers, formulating a super-hydrophobic aerogel which indicated high hydrophobicity and oil sorption efficiency. The natural-based aerogel was able to sorb the motor oil within 7 min, reaching a maximum adsorption capacity of ~95 g/g. Shi et al. [86] fabricated natural-based aerogels with lamellar structures (Figure 5c) using pomelo peel powder. The authors used MTMS to modify the initial pomelo peel-based sponge aerogels (PSA) to enhance their hydrophobicity. The new HPSA (hydrophobic pomelo peel-based sponge aerogel) indicated good oil/water sorption selectivity, with a crude oil sorption capacity of ~48 g/g. Additionally, the oil that adsorbed onto the aerogel can be retrieved by simple mechanical squeezing, maintaining a high oil sorption capacity upon several sorption cycles, indicating excellent recyclability [86]. It is noteworthy that the key advantage of the studied material was its simplicity, inexpensiveness, and eco-friendliness. The simplicity of the method is shown in Figure 9.

Zhang et al. (2014) used MTMS to modify cellulose for the preparation of a superhydrophobic sponge. MTMS was added into the cellulose solution and refluxed for 2 h under acidic conditions (pH = 4). Subsequently, the cellulosic aerogel was fabricated via freeze-drying. Its density (17.3 mg/cm^3^) and high porosity (99%) were easily manipulated by the freeze-drying of water suspensions of nano-fibrillated cellulose (NFC). The prepared aerogel had a superior hydrophobic/lipophilic surface with a very high recovery rate even after ten oil absorption/desorption cycles, and the absorption capacity was as high as 102 g/g. The aerogels modified with MTMS (Figure 9) possessed a layered structure, the surface shape was unchanged, and the pore size was kept constant [86].

**Figure 10:**
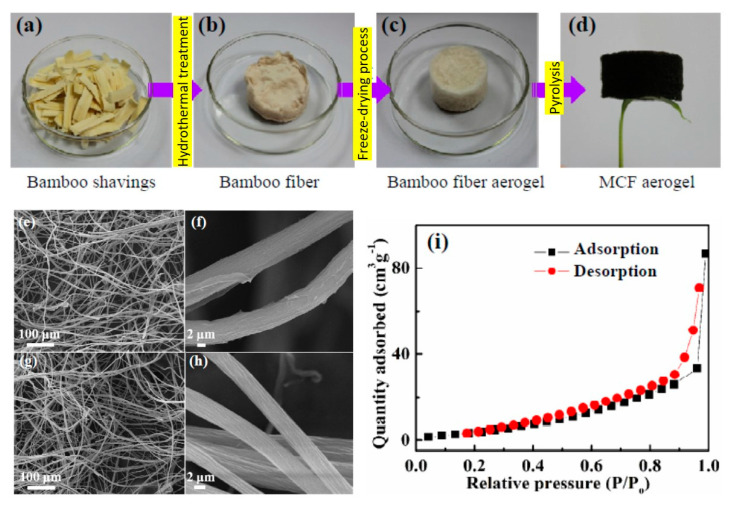
(**a**–**d**) Preparation of MCF aerogel, (**e**,**f**) bamboo fiber aerogel; (**g**,**h**) MCF aerogel, and (**i**) nitrogen adsorption–desorption isotherm of MCF aerogel (adapted and modified from [67] with permission from RSC).

Yang et al. [67] fabricated MCF (multi-functional carbon fiber) aerogel by a simple method using natural bamboo chopsticks as raw material (Figure 8). The SEM images (Figure 10e–h) revealed that a porous structure could be observed among the neighboring fibers (Figure 10e), with the diameter of fibers ranging from 8 to 10 µm (Figure 10f). Moreover, after the pyrolysis procedure, the porous structure was maintained (Figure 10g), and the pyrolyzed MCF aerogel displayed a hollow shape and smoother surface with a smaller diameter (Figure 10h). Nitrogen adsorption–desorption isotherm of multi-functional carbon fiber aerogel is presented in Figure 10i, indicating the formation of multilayer adsorption of nitrogen in MCF aerogel [67]. The MCF aerogel shows a moderate oil uptake capacity (~80 g/g) in comparison with some ultra-flyweight synthetic aerogels. Nevertheless, the material can be recycled easily using distillation, combustion, or squeezing, due to its porous and hydrophobic nature. Moreover, it shows high thermal and mechanical stability [67].

**Figure 11:**
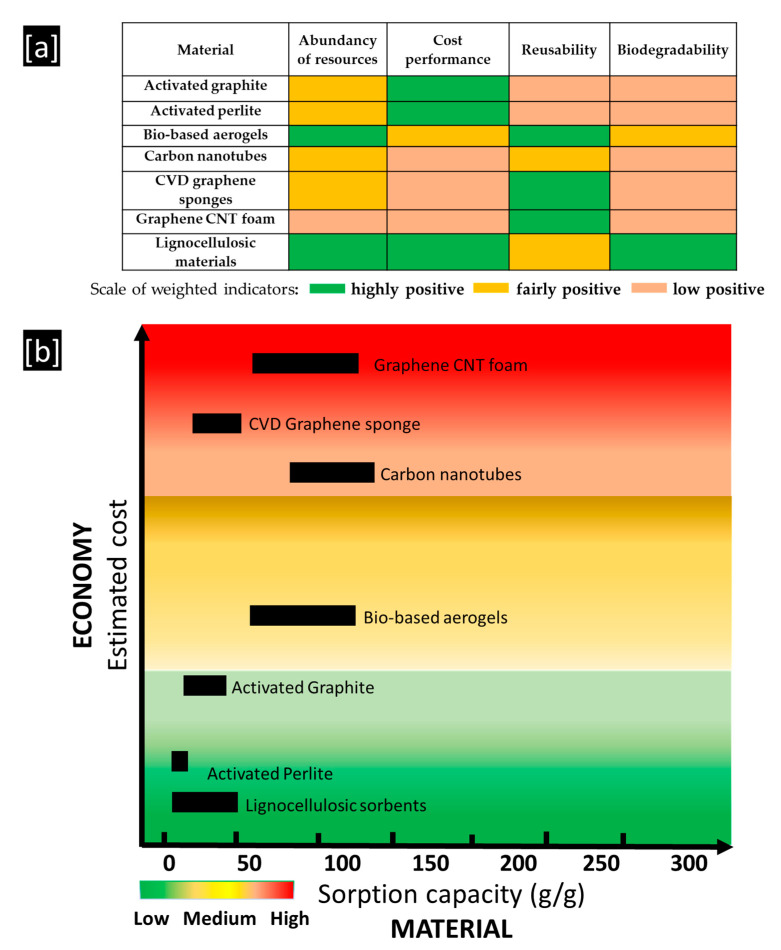
(**a**,**b**) Evaluation of materials as a process of an integrated approach based on the terms of material–environment–economy (authors’ own study, data from [99,100,101,102,103,104,105,106]).

Based on the analysis above, the main research outcomes are critically represented in Figure 11a below. In this figure, the outcomes from the literature review were evaluated in terms of weighted indicators. It can be concluded that the lignocellulosic materials and bio-based aerogels are mostly advantageous from an environmental point of view and in the context of cost performance (Figure 11a). Moreover, according to the assumptions of Yang et al. [67], the sorption capacity and estimated cost (with considerations on availability of resources, fabrication process, applicability of materials) of lignocellulosic materials were compared with other inorganic minerals and ultra-flyweight synthetic aerogels (Figure 11b). It was apparent that the lignocellulosic materials displayed higher sorption capacity than activated perlite and graphite, and bio-based aerogels exhibited similar sorption to the synthetic aerogel/foam, i.e., carbon nanotubes (CNTs) and graphene CNT foam (Figure 11b). Moreover, the fabrication method of bio-based aerogels was much simpler and their precursors, i.e., bamboo fibers or pomelo peel powder and others, are abundant and low-cost (Figure 11a,b).

**Table 1:** Oil sorption capacity of selected lignocellulosic materials.

Sorbent	Form of Sorbent	Modification Method	Oil Sorption Method	Adsorption Capacity	Type of Oil	Recovery	Refs
Banana peel	powder	raw	ASTM *	5–7	Crude oil	20 cycles	[24]
Sawdust	fiber	raw	-	4.1–6.4	Crude oil	n/a	[32]
Coir fiber	fiber	raw	-	1.8–5.4	Crude oil	n/a	[32]
Luffa	fiber	raw	-	1.9–4.6	Crude oil	n/a	[32]
Luffa	fiber	raw	-	14	Diesel oil	3 cycles	[107]
Orange peel	powder	raw	-	3–5	Crude, diesel, and used engine oil	5 cycles	[34]
Barley straw	powder	raw	-	6.5–12	Crude oil	3 cycles	[44]
Jute	fiber	raw	ASTM	2.6	Engine oil	n/a	[50]
Cotton	fiber	raw	ASTM	18.4–22.5	Used lube oil	5 cycles	[80]
Cotton	fiber	raw	ASTM	30.5	Motor oil	n/a	[108]
Sugarcane bagasse		raw	-	10.5–19.9	Diesel oil	n/a	[103]
Kapok	fiber	raw	-	19.4–49.9	Diesel, crude, engine oil, used engine oil	n/a	[103]
Kapok	fiber	raw	ASTM	36–45	Engine, diesel, and hydraulic oil	4 cycles	[109]
Kapok	fiber	raw	ASTM	50	Engine oil	15 cycles	[110]
Kapok	fiber	raw	ASTM	38	Diesel oil	8 cycles	[104]
Walnut shell	granular	raw	-	0.30–0.58	Mineral oil, vegetable oil and Bright-Edge oil	n/a	[111]
Rice husk	powder	raw		6.2		n/a	[112]
Rice husk	powder	pyrolysis	-	3.7–9.2	Diesel oil, gasoline, light crude, motor oil, heavy, crude oil	n/a	[58]
Rice husk	powder	pyrolysis	-	15	Crude oil		[47]
Rice husk	powder	pyrolysis	-	2.7–6.2	Diesel oil, crude oil		[59]
Rice husk	powder	pyrolysis	JIS technical standards	6	Heavy crude oil	n/a	[112]
Barley straw	fiber	pyrolysis	ASTM	7.6–9.2	Diesel, heavy crude oil	2 cycles	[62]
Rice husk	powder	mercerization	-	4–19	Marine oil	n/a	[75]
Soybean	powder	mercerization	ASTM	5	Crude oil	n/a	[49]
Cattail	fiber	mercerization	ASTM	4	Motor oil	n/a	[49]
Oil palm empty fruit bunch	granular	acetylation	-	7	Crude oil	n/a	[46]
Cocoa pods	fiber	acetylation	-	8	Crude oil	n/a	[46]
Jute	fiber	acetylation	ASTM	21.8	Engine oil	3 cycles	[50]
bamboo	aerogel	aerogel	-	38	Engine oil	5 cycles	[105]
Pomelo peel	aerogel	aerogel	-	5	Castor oil	5 cycles	[106]
Waste paper	aerogel	Aerogel with MTMS	-	24.4	Crude oil	5 cycles	[95]
Pomelo peel	aerogel	Aerogel with MTMS	-	49.8	Crude oil	10 cycles	[86]

* American society for testing and materials (ASTM)

## 5. Conclusions

The study of the applicability of raw lignocellulosic sorbents for oil spill cleanup is driven by their abundance, inexpensiveness, non-toxicity, reusability, and biodegradability. The drawbacks of these materials are low hydrophobicity, compromised oil sorption performance, and buoyancy properties. These properties can be enhanced by modification with specific agents. Thus, in the present study, various modification methods for hydrophobicity improvement of lignocellulosic materials have been reviewed. Material performance indicators, i.e., sorption capacity and reusability, as a function of specific application and testing conditions were examined in detail. Based on the review outcomes, we can conclude that the effectiveness of lignocellulosic sorbents classifies them among the best and most eco-friendly materials compared to other synthetic sorbents. Lignocellulosic sorbents are abundant in nature and their use for oil spill cleanup has been attracting significant attention lately. Some of the natural-based materials (bio-aerogels) have shown better or similar oil sorption capacities to commercial synthetic sorbents. Bio-based aerogels can be recycled easily using distillation, combustion, or squeezing, due to their porous and hydrophobic nature. However, in many studied aerogels, oil recovery methods (distillation and squeezing) tend to decrease the absorption capacity during several cycles, leading to degradation of the sorbent’s structure. On the other hand, synthesis of aerogels can be very environmentally friendly when the biomaterial is dispersed in water, freeze-dried, and, in some cases, also pyrolyzed during synthesis, avoiding additional chemicals or reagents. The multi-functional character of bio-based aerogels as depicted by their (a) superhydrophobicity, (b) satisfactory oil uptake efficiency, (c) recyclability, and (d) low-cost make them potential eco-sorbents for oil spill cleanup.
